# Identification of key miRNAs and genes associated with stomach adenocarcinoma from The Cancer Genome Atlas database

**DOI:** 10.1002/2211-5463.12365

**Published:** 2018-01-02

**Authors:** Jixi Liu, Fang Liu, Yanfen Shi, Huangying Tan, Lei Zhou

**Affiliations:** ^1^ Department of Gastroenterology China‐Japan Friendship Hospital Beijing China; ^2^ Department of Pathology China‐Japan Friendship Hospital Beijing China; ^3^ Department of Integrative Oncology China‐Japan Friendship Hospital Beijing China; ^4^ Department of General Surgery China‐Japan Friendship Hospital Beijing China

**Keywords:** differentially expressed genes, differentially expressed miRNA, signal pathway, stomach adenocarcinoma

## Abstract

Stomach adenocarcinoma (STAD) is the second leading cause of cancer death and a fuller understanding of its molecular basis is needed to develop new therapeutic targets. miRNA and mRNA data were downloaded from The Cancer Genome Atlas database, and the differentially expressed miRNAs and genes were identified. The target genes of differentially expressed miRNAs were screened by prediction tools. Furthermore, the biological function of these target genes was investigated. Several key miRNAs and their target genes were selected for validation using quantitative real‐time polymerase chain reaction (qRT‐PCR). The Gene Expression Omnibus (GEO) dataset was used to verify the expression of selected miRNAs and target genes. The diagnostic value of identified miRNAs and genes was accessed by receiver operating characteristic analysis. A total of 1248 differentially expressed genes were identified in STAD. Additionally, nine differentially expressed miRNAs were identified and 160 target genes of these nine miRNAs were identified via target gene detection. Interestingly, they were remarkably enriched in the calcium signaling pathway and bile secretion. qRT‐PCR confirmed the expression of several key miRNAs and their target genes. The expression levels of hsa‐miR‐145‐3p, hsa‐miR‐145‐5p, *ADAM12*,*ACAN*,*HOXC11* and *MMP11* in the GEO database were compatible with the bioinformatics results. hsa‐miR‐139‐5p, hsa‐miR‐145‐3p and *MMP11* have a potential diagnostic value for STAD. Differential expression of the mature form of miRNAs (hsa‐miR‐139‐5p, hsa‐miR‐145‐3p, hsa‐miR‐145‐5p and hsa‐miR‐490‐3p) and genes including *ADAM12*,*ACAN*,*HOXC11* and *MMP11* and calcium and bile secretion signaling pathways may play important roles in the development of STAD.

**Abbreviations**
AUC: area under the curve
DAVID: Database for Annotation, Visualization and Integrated Discovery
DEG: differentially expressed gene
FDR: false discovery rate
GEO: Gene Expression Omnibus
GO: gene ontology
KEGG: Kyoto Encyclopedia of Genes and Genomes
MTI: miR–target interaction
qRT‐PCR: quantitative real‐time polymerase chain reaction
ROC: receiver operating characteristic
STAD: stomach adenocarcinoma
TCGA: The Cancer Genome Atlas

Stomach carcinoma is a leading cause of cancer death 1. Stomach adenocarcinoma (STAD) is the most common form of malignant stomach carcinoma and generally affects older people (50–70 years of age) 2. According to the Lauren classification, there are two main types of STAD defined as the diffuse type and the intestinal type. Some risk factors are involved in STAD including smoking, high‐salt diet, chronic gastritis and *Helicobacter pylori* infection 3. Up to now, the principal treatment of STAD has been gastrectomy accompanied by chemotherapy and radiation therapy. Despite advances in the treatment of STAD, the 5‐year survival rate is 5–15% 4. Therefore, understanding the pathogenesis of STAD and searching for new therapeutic targets of STAD are urgent issues.

Recently, several studies have improved our understanding of the molecular mechanisms and signaling pathways underlying tumorigenesis in STAD. For example, some tyrosine kinase receptors, including ERBB2, EGFR, FGFR2 and MET, are activated, which leads to tumorigenesis in STAD 5. Moreover, the phosphoinositide‐3‐kinase–Akt signaling pathway is activated and results in aggressive proliferation of STAD tumors 6, 7, 8.

The Cancer Genome Atlas (TCGA) database is an application platform for genome analysis consisting of large‐sample genome sequencing data analysis for 33 types of cancers, including STAD 9. miRNAs function as post‐transcriptional regulators that can repress translation or promote degradation or cleavage of complementary target mRNA sequences 10, and moreover, miRNAs have emerged as key players in the pathogenesis of STAD 11. In this study, we downloaded the miRNA and mRNA data for STAD from TCGA database and identified several differentially expressed miRNAs and a number of differentially expressed genes (DEGs). Then, we obtained the target genes of these differentially expressed miRNAs and analyzed their biological function. We used the quantitative real‐time polymerase chain reaction (qRT‐PCR) method to validate some bioinformatics analysis results. The Gene Expression Omnibus (GEO) was used to verify the expression of selected miRNAs and target genes. Finally, the diagnostic value of the identified miRNAs and genes was accessed by receiver operating characteristic (ROC) analysis. These findings may enable us to understand the progression and development of STAD.

## Materials and methods

### Identification of differentially expressed miRNAs and genes

From TCGA database (http://cancergenome.nih.gov), we downloaded miRNA (IlluminaHiSeq_miRNASeq) data of 84 samples (42 case samples and 42 normal samples) and mRNA (IlluminaHiSeq_RNASeqV2) data of 64 samples including 32 cases and 32 normal samples. Differential expression between normal and case samples was assessed using the R‐bioconductor package deseq 2, and the *P*‐value was calculated. Multiple hypothesis testing was performed via the Benjamini–Hochberg procedure. Differentially expressed miRNAs were screened with the threshold of the false discovery rate (FDR) < 0.001, log2 fold change > 2 and mean base > 100. The DEGs were screened with the threshold of FDR < 0.001 and absolute value of log2 fold change > 2.

### Target gene detection of differentially expressed miRNAs

Six miRNA‐target prediction tools including rna22 (https://cm.jefferson.edu/rna22v2.0/), miranda (http://www.microrna.org/), mirdb (http://mirdb.org/miRDB/), mirwalk (http://www.umm.uni-heidelberg.de/apps/zmf/mirwalk/index.html), pictar2 (http://pictar.mdc-berlin.de/) and targetscan (http://www.targetscan.org/) were utilized to predict target genes of differentially expressed miRNAs. Target genes predicted by more than four algorithms or verified by experiments provided by the miRWalk database were screened out. Finally, the regulatory network between differentially expressed miRNAs and target genes was established, which was visualized using cytoscape software 12.

### Functional annotation of target genes

To further investigate the biological function, all DEGs and target genes of differentially expressed miRNAs were analyzed in the context of several databases such as Gene Ontology (GO) functional categories and the Kyoto Encyclopedia of Genes and Genomes (KEGG) biochemical pathway using the Database for Annotation, Visualization and Integrated Discovery (DAVID).

### qRT‐PCR validation

Among the identified differentially expressed miRNAs and their target genes, we selected miRNAs and genes with expression significance for and association with STAD. Depending on this criterion, hsa‐miR‐139‐5p, hsa‐miR‐145‐3p, hsa‐miR‐145‐5p, hsa‐miR‐490‐3p and their target genes *ADAM12*,* ACAN*,* HOXC11* and *MMP11* were selected for validation. Three tumor tissues and three para‐carcinoma tissues from participating individuals were obtained immediately after surgery. The collected tissues were frozen in liquid nitrogen for further RNA extraction. All participating individuals provided informed consent with the approval of the China‐Japan Friendship Hospital.

Total RNA of the tissue samples was extracted using the TRIzol^®^ Reagent (Invitrogen, Carlsbad, CA, USA) according to the manufacturer's protocols. One microgram of RNA was applied to synthesize DNA by SuperScript^®^ III Reverse Transcriptase (Invitrogen). Then real‐time PCR was performed in an ABI 7500 real‐time PCR system with SYBR^®^ Green PCR Master Mix (Invitrogen). To confirm their reliability and validity, U6 and 18S rRNA were selected as the endogenous standards. All reactions were carried out in triplicate and relative gene expressions were analyzed by the 2^−ΔΔ*C*t^ method. The primer sequence is shown in Table 1.

**Table 1:** The primer sequence in the qRT‐PCR

Primer	Sequence (5′ to 3′)
*ADAM12*	Forward: AAGACCTTGATACGACTGCTGTTT
	Reverse: GACTGGGGCTGAGGGACATT
*ACAN*	Forward: GTCACACCTGAGCAGCATCGT
	Reverse: CTGGTAGTCTTGGGCATTGTTGT
*HOXC11*	Forward: GGCTGAGGAGGAGAACACAAATC
	Reverse: GCCGCTTCTCTTTGTTGATATACAC
*MMP11*	Forward: CCCGCAACCGACAGAAGAG
	Reverse: GGCGTCACATCGCTCCATAC
18S rRNA	Forward: GTAACCCGTTGAACCCCATT
	Reverse: CCATCCAATCGGTAGTAGCG
hsa‐miR‐139‐5p	Forward: TCTACAGTGCACGTGTCTCCAGT
hsa‐miR‐145‐3p	Forward: GGATTCCTGGAAATACTGTTCT
hsa‐miR‐145‐5p	Forward: GTCCAGTTTTCCCAGGAATC
hsa‐miR‐490‐3p	Forward: CAACCTGGAGGACTCCATGCT
U6	Forward: CTCGCTTCGGCAGCACA
	Reverse: AACGCTTCACGAATTTGCGT

### Validation of the expression of miRNAs and target genes by GEO

The GEO (http://www.ncbi.nlm.nih.gov/geo) database was used to validate the expression of selected miRNAs and targeted genes. We compared the expression levels of miRNAs and targeted genes between STAD cases and adjacent non‐tumor controls and the difference of expression levels were displayed as box‐plots.

### ROC analyses

Using the proc package in the R language we, performed the ROC analyses to assess the diagnostic value of selected miRNAs and target genes. The area under the curve (AUC) under binomial exact confidence interval was calculated and the ROC curve was generated.

## Results

### Differentially expressed miRNAs and genes

**Figure 1:**
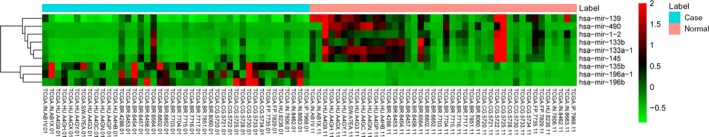
Heat map of differentially expressed precursor form of miRNAs in STAD.

**Figure 2:**
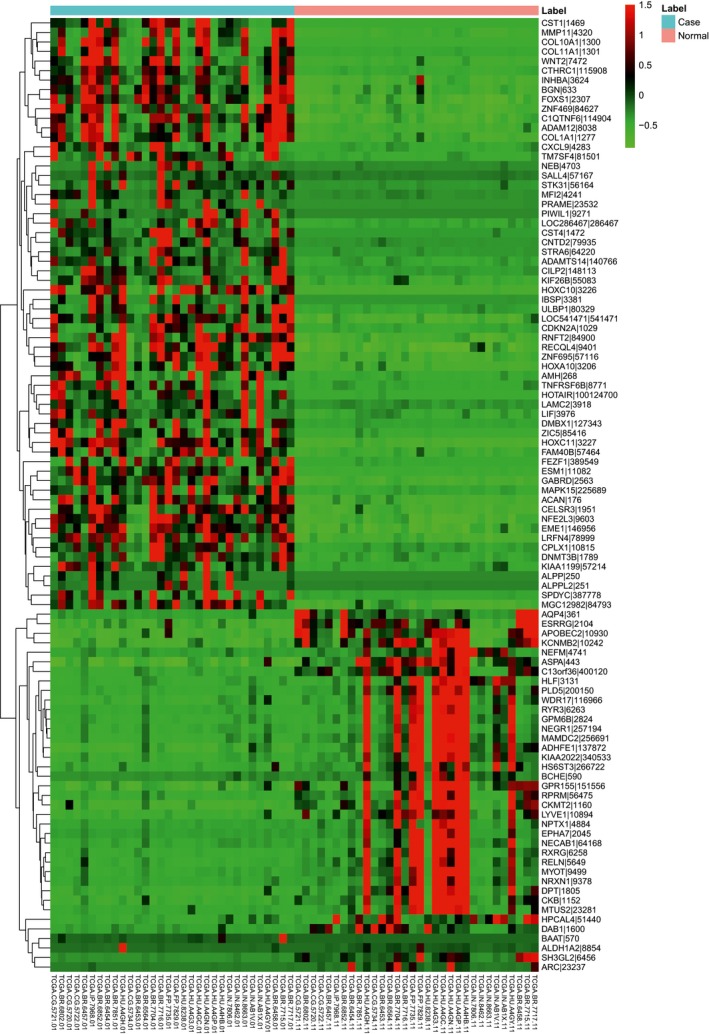
Heat map of top 100 differentially expressed genes in STAD.

In this study, we found that nine miRNAs (three up‐regulated and six down‐regulated) and 1248 genes (371 up‐regulated and 877 down‐regulated) were differentially expressed in STAD. Table 2 shows all differentially expressed miRNAs (precursor form) including hsa‐mir‐139, hsa‐mir‐196a‐1, hsa‐mir‐196b, hsa‐mir‐133a‐1, hsa‐mir‐1‐2, hsa‐mir‐145, hsa‐mir‐135b, hsa‐mir‐133b and hsa‐mir‐490. The top 20 DEGs are listed in Table 3. Additionally, heat maps of these differentially expressed precursor form of miRNAs and genes are shown in Figs 1 and 2, respectively.

**Table 2:** The differentially expressed precursor form of miRNAs in STAD

miRNA	Mean base	Log2 fold change	Standard error	Wald statistic	*P*	*P* adj	Up/down
hsa‐mir‐139	528.1534	−2.55383	0.202603	−12.6051	1.98 × 10^−36^	3.51 × 10^−34^	Down
hsa‐mir‐196a‐1	392.8496	4.36323	0.345863	12.6155	1.73 × 10^−36^	3.51 × 10^−34^	Up
hsa‐mir‐196b	1049.324	4.091521	0.342584	11.94312	7.05 × 10^−33^	8.35 × 10^−31^	Up
hsa‐mir‐133a‐1	900.0538	−3.34948	0.30633	−10.9342	7.91 × 10^−28^	7.02 × 10^−26^	Down
hsa‐mir‐1‐2	1498.213	−3.23485	0.331376	−9.76188	1.64 × 10^−22^	8.32 × 10^−21^	Down
hsa‐mir‐145	48448.02	−2.37286	0.249126	−9.52473	1.65 × 10^−21^	7.34 × 10^−20^	Down
hsa‐mir‐135b	127.1043	2.927057	0.315644	9.273278	1.81 × 10^−20^	6.41 × 10^−19^	Up
hsa‐mir‐133b	127.2602	−2.76184	0.307681	−8.97632	2.80 × 10^−19^	8.28 × 10^−18^	Down
hsa‐mir‐490	255.5693	−3.83958	0.488705	−7.85665	3.95 × 10^−15^	7.37 × 10^−14^	Down

**Table 3:** Top 20 DEGs in STAD

mRNA	Mean base	Log2 fold change	Standard error	Wald statistic	*P*	FDR	Up/down
CST1|1469	1950.411	7.706956	0.456963	16.86562	8.06 × 10^−64^	1.56 × 10^−59^	Up
COL10A1|1300	628.3591	6.468628	0.398018	16.25211	2.16 × 10^−59^	2.09 × 10^−55^	Up
ESM1|11082	164.0381	4.772024	0.307322	15.52774	2.25 × 10^−54^	1.45 × 10^−50^	Up
GABRD|2563	54.36653	3.639088	0.279383	13.02546	8.77 × 10^−39^	4.24 × 10^−35^	Up
HOXC10|3226	356.3751	6.328307	0.500079	12.65462	1.05 × 10^−36^	4.08 × 10^−33^	Up
COL11A1|1301	372.4826	5.984012	0.488973	12.23792	1.95 × 10^−34^	6.28 × 10^−31^	Up
MMP11|4320	2090.436	3.807886	0.314897	12.09247	1.16 × 10^−33^	3.20 × 10^−30^	Up
LYVE1|10894	921.3208	−3.57139	0.304514	−11.7282	9.14 × 10^−32^	2.21 × 10^−28^	Down
HOTAIR|100124700	45.54065	5.787221	0.502089	11.52628	9.73 × 10^−31^	2.09 × 10^−27^	Up
CTHRC1|115908	845.8077	3.275777	0.284703	11.50593	1.23 × 10^−30^	2.38 × 10^−27^	Up
LRFN4|78999	1583.359	2.39677	0.211807	11.3158	1.10 × 10^−29^	1.93 × 10^−26^	Up
C1QTNF6|114904	519.9666	2.003764	0.177663	11.27842	1.68 × 10^−29^	2.70 × 10^−26^	Up
PIWIL1|9271	134.2764	6.952432	0.621324	11.18971	4.58 × 10^−29^	6.81 × 10^−26^	Up
SH3GL2|6456	62.35987	−4.47119	0.407905	−10.9613	5.86 × 10^−28^	8.09 × 10^−25^	Down
CILP2|148113	126.4808	3.88935	0.356923	10.89688	1.19 × 10^−27^	1.54 × 10^−24^	Up
HOXC11|3227	157.2612	4.742368	0.449461	10.55122	5.01 × 10^−26^	6.06 × 10^−23^	Up
STRA6|64220	295.6888	4.633958	0.439962	10.53262	6.11 × 10^−26^	6.95 × 10^−23^	Up
ALPP|250	75.03801	6.927139	0.658164	10.52494	6.63 × 10^−26^	7.12 × 10^−23^	Up
CELSR3|1951	616.3266	2.799618	0.267519	10.46512	1.25 × 10^−25^	1.27 × 10^−22^	Up
MAPK15|225689	58.32309	3.731925	0.358736	10.40299	2.40 × 10^−25^	2.32 × 10^−22^	Up

### Identification of miRNA–target interactions

**Figure 3:**
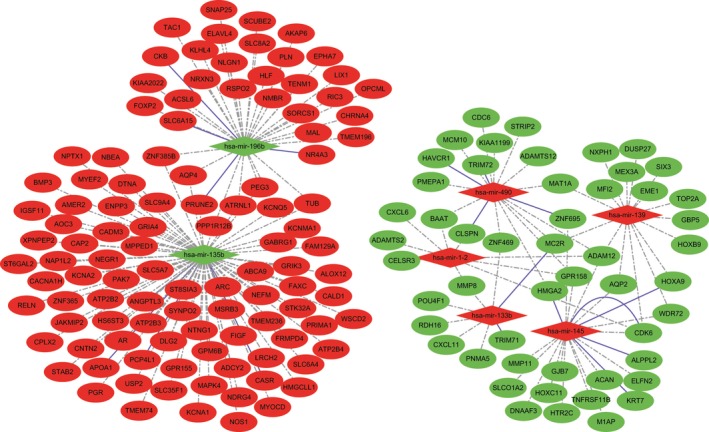
Regulatory network of precursor form of miRNAs and target genes in STAD. Rhombus and oval represent miRNA and target gene, respectively. Red and green colors represent up‐regulation and down‐regulation, respectively. The full blue lines indicate the miRNA–mRNA validation pairs and the dashed lines indicate the miRNA–mRNA prediction pairs.

In the present study, potential target genes of the differentially expressed precursor form of miRNAs were identified. We found 160 target genes with 270 miRNA–target pairs including 189 interaction pairs with miRNA up‐regulated and target genes down‐regulated and 81 interaction pairs with miRNA down‐regulated and target genes up‐regulated in STAD subjects. The established regulatory network of miRNA–targets is showed in Fig. 3. In addition, we further confirmed all of the miR–target interactions (MTIs) in TCGA data analysis using miRTarBAse. Our results showed that there was a total of 46 MTIs after the miRTarBAse analysis. However, we obtained a total of 187 MTIs by using the miRWalk database. Therefore, the MTIs in the miRWalk database were more than those in the miRTarBAse database. Taking the intersection of the miRTarBAse and miRWalk databases, a total of 22 common MTIs was identified. The original MTIs from the miRWalk and miRTarBAse databases are listed in Tables S1 and S2, respectively. A Venn diagram of MTIs in the groups of the miRTarBAse database *vs* the miRWalk database is shown in Fig. S1.

### Target genes enrichment analysis

**Figure 4:**
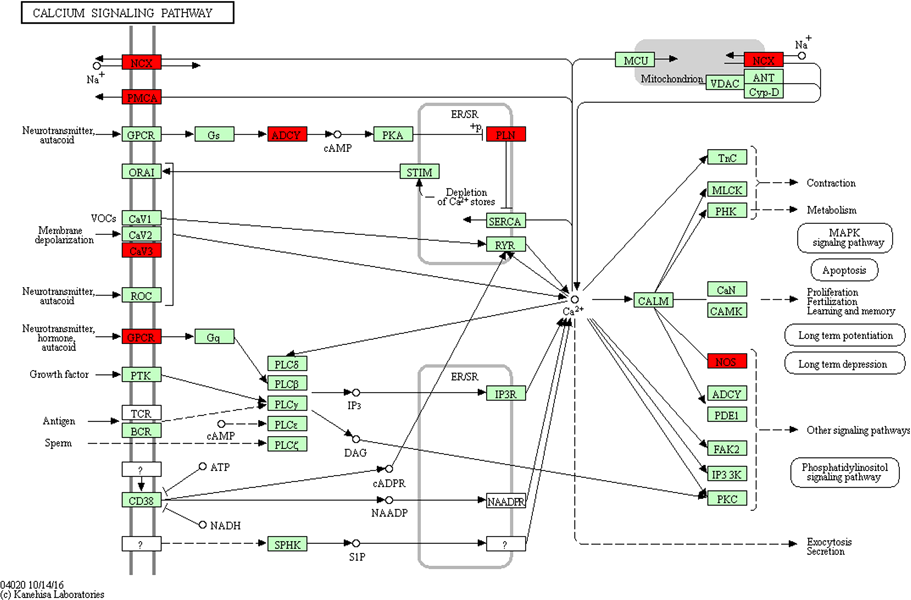
Target genes that were significantly enriched in calcium signaling pathway. The red rectangles represent the target genes that are enriched in calcium signaling pathway. The figure was obtained by the Kanehisa Laboratories.

**Figure 5:**
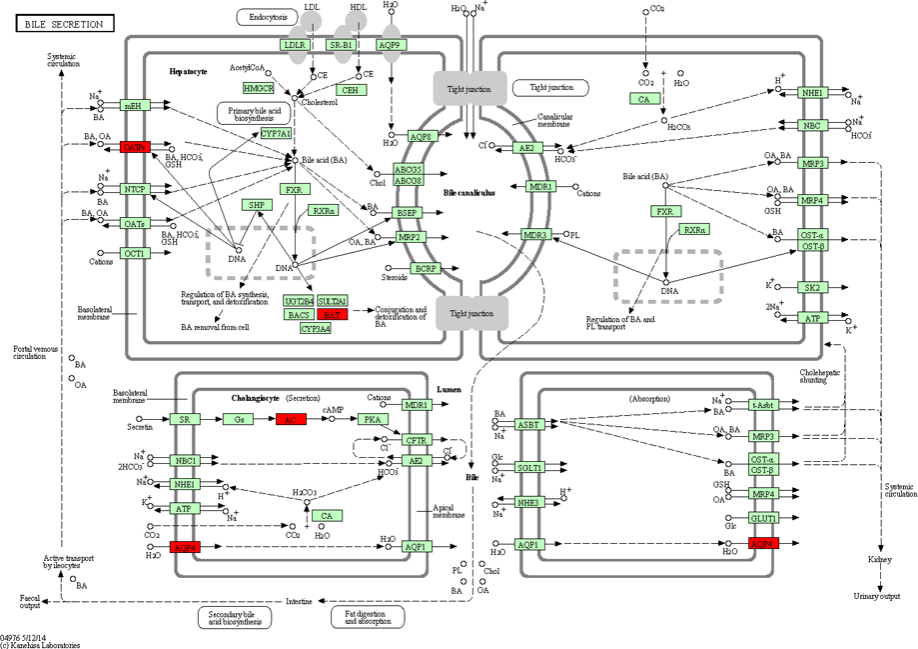
Target genes that were significantly enriched in bile secretion. The red rectangles were represented the target genes that are enriched in bile secretion. The figure was obtained by the Kanehisa Laboratories.

**Figure 6:**
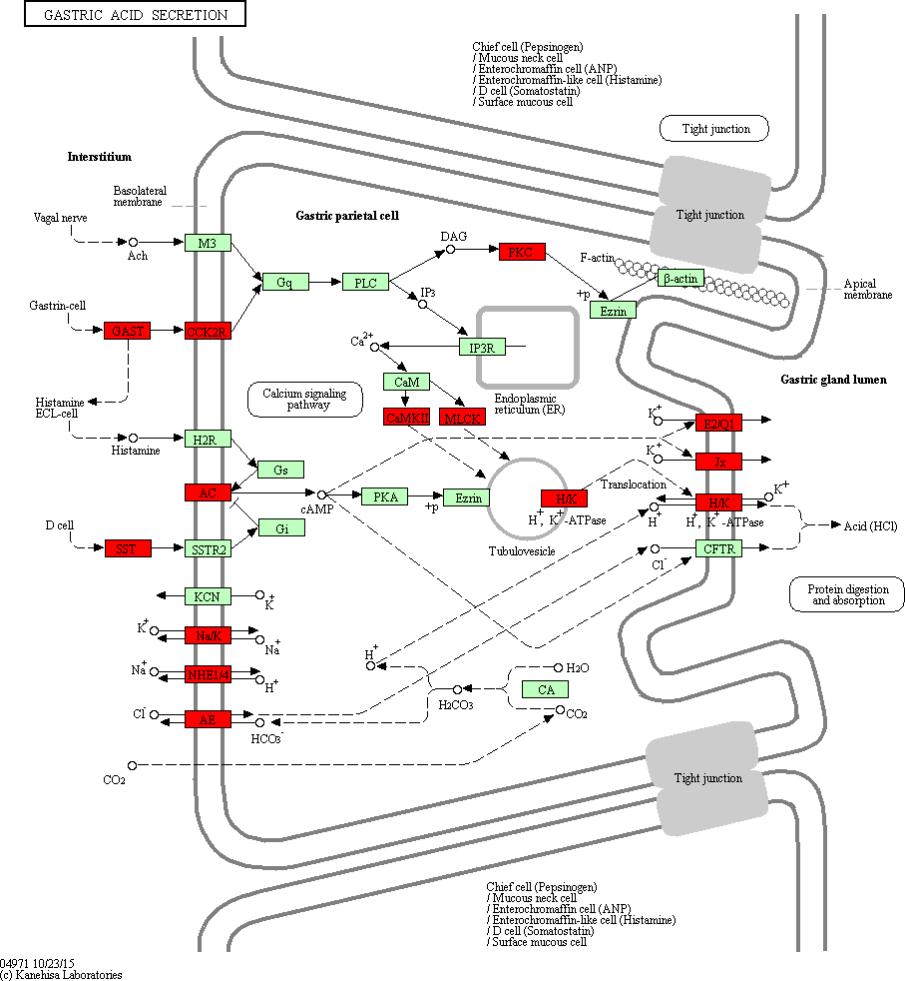
Target genes that were significantly enriched in gastric acid secretion. The red rectangles represent the target genes that are enriched in gastric acid secretion. The figure was obtained by the Kanehisa Laboratories.

Based on the DAVID database, we analyzed the biological function and pathways of these 160 target genes. Additionally, we also analyzed the function of all DEGs by KEGG. The enrichment of GO functional categories and KEGG biochemical pathways showed that these target genes were significantly enriched in calcium‐transporting ATPase activity (FDR = 0.001048), calmodulin binding (FDR = 0.002055), calcium signaling pathway (FDR = 7.97 × 10^−6^), salivary secretion (FDR = 9.13 × 10^−5^), pancreatic secretion (FDR = 0.00209), neuroactive ligand–receptor interaction (FDR = 0.004133), cell adhesion molecules (FDR = 0.003932), bile secretion (FDR = 0.003801) and vascular smooth muscle contraction (FDR = 0.018674). A GO analysis of these enriched target genes is shown in Table 4, a KEGG pathway analysis of these target genes is listed in Table 5 and the pathway maps in KEGG of calcium signaling pathway and bile secretion are shown in Figs 4 and 5, which are related to STAD. Interestingly, all DEGs were found to be enriched in the signal pathway of gastric acid secretion (Fig. 6), which also plays a role in STAD.

**Table 4:** GO function analysis of target genes

Item	Item details	Count	*P*	FDR	Genes
Molecular function					
GO:0046872	Metal ion binding	37	3.36 × 10^−9^	9.76 × 10^−7^	*ADCY2*,* MAT1A*,* MMP11*,* ALOX12*,* ATP2B3*,* TRIM72*,* XPNPEP2*,* MMP8*,* TRIM71*,* ATP2B4*,* ZNF469*,* EME1*,* KCNMA1*,* HMGCLL1*,* ADAMTS12*,* ADAM12*,* STK32A*,* NPTX1*,* ENPP3*,* FOXP2*,* NRXN3*,* PRUNE2*,* NOS1*,* MCM10*,* ALPPL2*,* ATP2B2*,* ZNF695*,* PGR*,* USP2*,* AR*,* NR4A3*,* ADAMTS2*,* ZNF385B*,* MSRB3*,* MFI2*,* MEX3A*,* RELN*
GO:0005388	Calcium‐transporting ATPase activity	3	7.23 × 10^−6^	0.001048	*ATP2B3*,* ATP2B4*,* ATP2B2*
GO:0043565	Sequence‐specific DNA binding (MF)	11	3.46 × 10^−5^	0.002009	*HLF*,* HOXA9*,* SIX3*,* HOXB9*,* FOXP2*,* HOXC11*,* POU4F1*,* PGR*,* TOP2A*,* AR*,* NR4A3*
GO:0005516	Calmodulin binding	6	5.67 × 10^−5^	0.002055	*ATP2B3*,* ATP2B4*,* CALD1*,* NOS1*,* ATP2B2*,* SLC8A2*
GO:0017022	Myosin binding	3	4.71 × 10^−5^	0.002275	*SLC6A4*,* CALD1*,* SNAP25*
GO:0005216	Ion channel activity	7	3.15 × 10^−5^	0.002282	*GRIK3*,* KCNMA1*,* KCNQ5*,* GRIA4*,* GABRG1*,* CHRNA4*,* KCNA2*
GO:0005515	Protein binding	38	5.65 × 10^−5^	0.002341	*CLSPN*,* DTNA*,* ACAN*,* ALOX12*,* HMGA2*,* SLC6A4*,* CELSR3*,* HOXA9*,* ATP2B4*,* HOXB9*,* EME1*,* KCNMA1*,* NTNG1*,* ADAM12*,* SORCS1*,* MYOCD*,* NEGR1*,* APOA1*,* MC2R*,* FRMPD4*,* CDC6*,* PLN*,* CASR*,* NOS1*,* MCM10*,* MAL*,* ZNF365*,* ATP2B2*,* PGR*,* TOP2A*,* USP2*,* AR*,* NEFM*,* MFI2*,* DLG2*,* KRT7*,* CDK6*,* SNAP25*
GO:0017075	Syntaxin‐1 binding	3	2.43 × 10^−5^	0.002347	*SLC6A4*,* CPLX2*,* SNAP25*
GO:0004872	Receptor activity	19	7.64 × 10^−5^	0.002463	*CELSR3*,* HTR2C*,* HAVCR1*,* GRIK3*,* GPR158*,* MC2R*,* GRIA4*,* NMBR*,* NRXN3*,* CASR*,* EPHA7*,* TNFRSF11B*,* PGR*,* CHRNA4*,* AR*,* NR4A3*,* STAB 2*,* IGSF11*,* NLGN1*
GO:0004222	Metalloendopeptidase activity	5	0.000108	0.00313	*MMP11*,* MMP8*,* ADAMTS12*,* ADAM12*,* ADAMTS2*
GO:0019899	Enzyme binding	6	0.000216	0.005705	*APOA1*,* PGR*,* TOP2A*,* PRIMA1*,* AR*,* AKAP6*
GO:0005249	Voltage‐gated potassium channel activity	4	0.000312	0.006971	*KCNMA1*,* KCNQ5*,* SNAP25*,* KCNA2*
GO:0005372	Water transmembrane transporter activity	2	0.000295	0.00712	*AQP2*,* AQP4*
GO:0005244	Voltage‐gated ion channel activity	5	0.000589	0.011389	*CACNA1H*,* KCNMA1*,* KCNQ5*,* KCNA1*,* KCNA2*
GO:0030165	PDZ domain binding	4	0.000562	0.011646	*ATP2B4*,* AQP2*,* ATP2B2*,* DLG2*
Biological process					
GO:0007268	Synaptic transmission	16	2.12 × 10^−11^	1.60 × 10^−8^	*ADCY2*,* DTNA*,* HTR2C*,* GRIK3*,* TAC1*,* KCNMA1*,* KCNQ5*,* GRIA4*,* NPTX1*,* GABRG1*,* CHRNA4*,* DLG2*,* KCNA1*,* SLC5A7*,* SNAP25*,* KCNA2*
GO:0055085	Transmembrane transport	18	5.43 × 10^−10^	2.04 × 10^−7^	*ADCY2*,* ATP2B3*,* CACNA1H*,* ATP2B4*,* KCNMA1*,* KCNQ5*,* SLC6A15*,* SLC9A4*,* APOA1*,* SLCO1A2*,* AQP2*,* ATP2B2*,* AQP4*,* SLC8A2*,* KCNA1*,* SLC5A7*,* GPR155*,* KCNA2*
GO:0006811	Ion transport	15	2.91 × 10^−8^	7.29 × 10^−6^	*CACNA1H*,* GRIK3*,* KCNMA1*,* KCNQ5*,* SLC6A15*,* SLC9A4*,* GRIA4*,* GABRG1*,* SLCO1A2*,* CHRNA4*,* MFI2*,* SLC8A2*,* KCNA1*,* SLC5A7*,* KCNA2*
GO:0007275	Multicellular organismal development	19	5.17 × 10^−8^	9.73 × 10^−6^	*HLF*,* MMP11*,* HMGA2*,* CELSR3*,* HOXA9*,* SIX3*,* TRIM71*,* HOXB9*,* NTNG1*,* ARC*,* HOXC11*,* FIGF*,* NDRG4*,* EPHA7*,* POU4F1*,* GPM6B*,* ST6GAL2*,* RELN*,* BMP3*
GO:0007155	Cell adhesion	13	1.45 × 10^−6^	0.000218	*OPCML*,* ACAN*,* CELSR3*,* ADAM12*,* NEGR1*,* CADM3*,* NRXN3*,* AOC3*,* STAB 2*,* IGSF11*,* CNTN2*,* RELN*,* NLGN1*
GO:0030168	Platelet activation	8	1.12 × 10^−5^	0.001409	*ATP2B3*,* ATP2B4*,* KCNMA1*,* APOA1*,* FIGF*,* NOS1*,* ATP2B2*,* SLC8A2*
GO:0007520	Myoblast fusion	3	1.41 × 10^−5^	0.001517	*CACNA1H*,* ADAM12*,* NOS1*
GO:0060748	Tertiary branching involved in mammary gland duct morphogenesis	2	5.94 × 10^−5^	0.005596	*PGR*,* AR*
GO:0030574	Collagen catabolic process	3	9.46 × 10^−5^	0.007122	*MMP11*,* MMP8*,* ADAMTS2*
GO:0006810	Transport	11	9.25 × 10^−5^	0.007743	*ATP2B3*,* CACNA1H*,* TRIM72*,* ATP2B4*,* GRIA4*,* NPTX1*,* SLC35F1*,* ATP2B2*,* AQP4*,* AR*,* ABCA9*
GO:0007420	Brain development	6	0.000161	0.011038	*CKB*,* SLC6A4*,* SIX3*,* EPHA7*,* ATP2B2*,* RELN*
GO:0001503	Ossification	4	0.000198	0.011493	*ACAN*,* MMP8*,* CASR*,* BMP3*
GO:0051346	Negative regulation of hydrolase activity	2	0.000197	0.012361	*APOA1*,* NOS1*
GO:0030879	Mammary gland development	3	0.000265	0.01329	*HOXA9*,* HOXB9*,* PGR*
GO:0042391	Regulation of membrane potential	3	0.000265	0.01329	*GRIK3*,* KCNMA1*,* CHRNA4*
Cellular component					
GO:0005886	Plasma membrane	57	1.47 × 10^−18^	2.16 × 10^−16^	*ADCY2*,* OPCML*,* DTNA*,* ATP2B3*,* FAM129A*,* SLC6A4*,* NBEA*,* CELSR3*,* HTR2C*,* XPNPEP2*,* GBP5*,* GRIK3*,* ATP2B4*,* PPP1R12B*,* TAC1*,* KCNMA1*,* KCNQ5*,* NTNG1*,* ACSL6*,* SLC6A15*,* SLC9A4*,* GPR158*,* ARC*,* ADAM12*,* NEGR1*,* CADM3*,* APOA1*,* MC2R*,* GRIA4*,* NMBR*,* CALD1*,* PMEPA1*,* CASR*,* EPHA7*,* ALPPL2*,* GABRG1*,* SLCO1A2*,* AQP2*,* ATP2B2*,* AOC3*,* PRIMA1*,* AQP4*,* CHRNA4*,* TUB*,* STAB 2*,* MFI2*,* IGSF11*,* GJB7*,* DLG2*,* SLC8A2*,* CNTN2*,* KCNA1*,* CAP2*,* SLC5A7*,* SNAP25*,* NLGN1*,* KCNA2*
GO:0030425	Dendrite	12	4.11 × 10^−11^	3.02 × 10^−9^	*ADCY2*,* GRIK3*,* NEGR1*,* GRIA4*,* CPLX2*,* EPHA7*,* ATP2B2*,* CHRNA4*,* AR*,* DLG2*,* RELN*,* NLGN1*
GO:0016021	Integral to membrane	50	1.70 × 10^−10^	8.34 × 10^−9^	*ADCY2*,* ATP2B3*,* CACNA1H*,* CELSR3*,* HTR2C*,* ELFN2*,* HAVCR1*,* GRIK3*,* KCNMA1*,* KCNQ5*,* ACSL6*,* SLC6A15*,* SLC9A4*,* GPR158*,* ADAM12*,* SORCS1*,* CADM3*,* GRIA4*,* PMEPA1*,* NRXN3*,* PLN*,* CASR*,* SLC35F1*,* GABRG1*,* SLCO1A2*,* AQP2*,* WSCD2*,* ATP2B2*,* TMEM74*,* AOC3*,* PRIMA1*,* ATRNL1*,* AQP4*,* CHRNA4*,* RIC3*,* GPM6B*,* ST8SIA3*,* RDH16*,* ST6GAL2*,* HS6ST3*,* IGSF11*,* GJB7*,* SLC8A2*,* KCNA1*,* ABCA9*,* SLC5A7*,* TMEM236*,* GPR155*,* KCNA2*,* TMEM196*
GO:0043025	Neuronal cell body	12	3.15 × 10^−10^	1.16 × 10^−8^	*TAC1*,* NEGR1*,* GRIA4*,* CALD1*,* CPLX2*,* EPHA7*,* ATP2B2*,* CHRNA4*,* DLG2*,* SLC8A2*,* CNTN2*,* SNAP25*
GO:0045202	Synapse	12	7.76 × 10^−9^	2.28 × 10^−7^	*DTNA*,* GRIK3*,* ARC*,* GRIA4*,* CPLX2*,* NOS1*,* GABRG1*,* PRIMA1*,* CHRNA4*,* DLG2*,* SNAP25*,* NLGN1*
GO:0044224	Juxtaparanode region of axon	4	2.66 × 10^−8^	6.51 × 10^−7^	*DLG2*,* CNTN2*,* KCNA1*,* KCNA2*
GO:0045211	Postsynaptic membrane	8	1.06 × 10^−6^	2.22 × 10^−5^	*GRIK3*,* ARC*,* GRIA4*,* EPHA7*,* GABRG1*,* CHRNA4*,* DLG2*,* NLGN1*
GO:0008076	Voltage‐gated potassium channel complex	6	3.53 × 10^−6^	5.77 × 10^−5^	*KCNMA1*,* KCNQ5*,* CNTN2*,* KCNA1*,* SNAP25*,* KCNA2*
GO:0005887	Integral to plasma membrane	17	3.34 × 10^−6^	6.14 × 10^−5^	*OPCML*,* SLC6A4*,* GRIK3*,* ATP2B4*,* SLC6A15*,* MC2R*,* NMBR*,* ENPP3*,* NRXN3*,* CASR*,* EPHA7*,* MAL*,* AQP4*,* STAB 2*,* MFI2*,* CNTN2*,* NLGN1*
GO:0016020	Membrane	38	6.88 × 10^−6^	0.000101	*ADCY2*,* CACNA1H*,* XPNPEP2*,* ELFN2*,* HAVCR1*,* KCNMA1*,* KCNQ5*,* SLC6A15*,* SORCS1*,* ENPP3*,* FIGF*,* NRXN3*,* PLN*,* SLC35F1*,* ALPPL2*,* SLCO1A2*,* AQP2*,* WSCD2*,* MAL*,* ATP2B2*,* TMEM74*,* ATRNL1*,* AQP4*,* CHRNA4*,* RIC3*,* GPM6B*,* ST8SIA3*,* RDH16*,* AKAP6*,* ST6GAL2*,* HS6ST3*,* SLC8A2*,* ABCA9*,* SLC5A7*,* TMEM236*,* GPR155*,* KCNA2*,* TMEM196*
GO:0030054	Cell junction	11	1.05 × 10^−5^	0.00014	*DTNA*,* GRIK3*,* ARC*,* GRIA4*,* GABRG1*,* PRIMA1*,* CHRNA4*,* GJB7*,* DLG2*,* SNAP25*,* NLGN1*
GO:0031225	Anchored to membrane	6	1.17 × 10^−5^	0.000144	*OPCML*,* XPNPEP2*,* NEGR1*,* ALPPL2*,* MFI2*,* CNTN2*
GO:0043197	Dendritic spine	5	1.50 × 10^−5^	0.000169	*ARC*,* FRMPD4*,* CALD1*,* NOS1*,* SLC8A2*
GO:0005578	Proteinaceous extracellular matrix	7	4.58 × 10^−5^	0.000481	*ACAN*,* MMP11*,* MMP8*,* ADAMTS12*,* TNFRSF11B*,* ADAMTS2*,* RELN*
GO:0005737	Cytoplasm	43	4.99 × 10^−5^	0.000489	*ADCY2*,* DTNA*,* CKB*,* KIAA1199*,* ALOX12*,* FAM129A*,* NBEA*,* HTR2C*,* HOXA9*,* TRIM71*,* PPP1R12B*,* BAAT*,* ARC*,* NMBR*,* CALD1*,* CDC6*,* FOXP2*,* HOXC11*,* NDRG4*,* SYNPO2*,* CPLX2*,* PRUNE2*,* NOS1*,* MCM10*,* AQP2*,* ZNF365*,* ATP2B2*,* PGR*,* TOP2A*,* AQP4*,* USP2*,* AR*,* TUB*,* PAK7*,* AKAP6*,* STAB 2*,* DLG2*,* MEX3A*,* KRT7*,* CDK6*,* SNAP25*,* RELN*,* KLHL4*

**Table 5:** The KEGG pathway analysis of target genes

Item	Item details	Count	*P*	FDR	Genes
Kegg:04020	Calcium signaling pathway	9	1.03 × 10^−7^	7.97 × 10^−6^	*SLC8A2*,* ADCY2*,* ATP2B4*,* CACNA1H*,* HTR2C*,* ATP2B2*,* PLN*,* NOS1*,* ATP2B3*
Kegg:04970	Salivary secretion	6	2.37 × 10^−6^	9.13 × 10^−5^	*ADCY2*,* KCNMA1*,* ATP2B4*,* ATP2B2*,* NOS1*,* ATP2B3*
Kegg:04972	Pancreatic secretion	5	8.14 × 10^−5^	0.00209	*ADCY2*,* KCNMA1*,* ATP2B4*,* ATP2B2*,* ATP2B3*
Kegg:04080	Neuroactive ligand–receptor interaction	7	0.000215	0.004133	*NMBR*,* MC2R*,* HTR2C*,* GABRG1*,* GRIK3*,* GRIA4*,* CHRNA4*
Kegg:04514	Cell adhesion molecules	5	0.000255	0.003932	*NRXN3*,* CNTN2*,* NLGN1*,* CADM3*,* NEGR1*
Kegg:04976	Bile secretion	4	0.000296	0.003801	*ADCY2*,* AQP4*,* SLCO1A2*,* BAAT*
Kegg:04270	Vascular smooth muscle contraction	4	0.001698	0.018674	*ADCY2*,* KCNMA1*,* CALD1*,* PPP1R12B*

### qRT‐PCR verification of selected miRNA and genes

**Figure 7:**
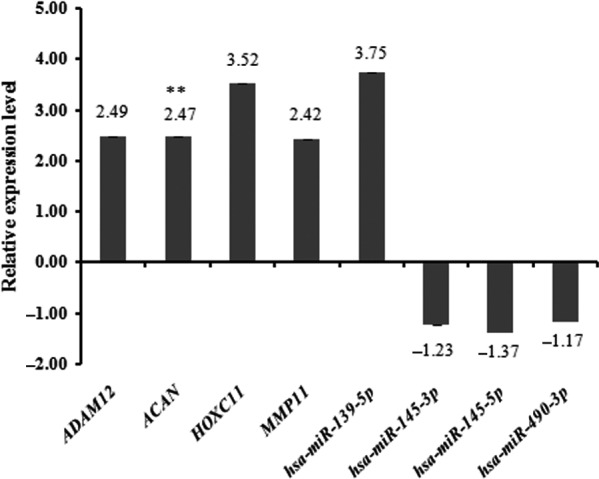
Validation of differentially expressed mature forms of miRNAs and mRNAs in the STAD tissues by qRT‐PCR. **P < 0.01.

To confirm the validity and reliability of bioinformatics results, we selected and assessed the expression levels of four key mature forms of miRNAs (hsa‐miR‐139‐5p, hsa‐miR‐145‐3p, hsa‐miR‐145‐5p and hsa‐miR‐490‐3p) and their target genes including *ADAM12*,* ACAN*,* HOXC11* and *MMP11* (Fig. 7). The results showed that hsa‐miR‐145‐3p, hsa‐miR‐145‐5p and hsa‐miR‐490‐3p showed down‐regulation, and their target genes (*ADAM12*,* ACAN*,* HOXC11* and *MMP11*) showed up‐regulation, consistent with the bioinformatics results. However, the expression of hsa‐miR‐139‐5p was inconsistent with the bioinformatics result.

### Validation the expression of miRNAs and target genes

**Figure 8:**
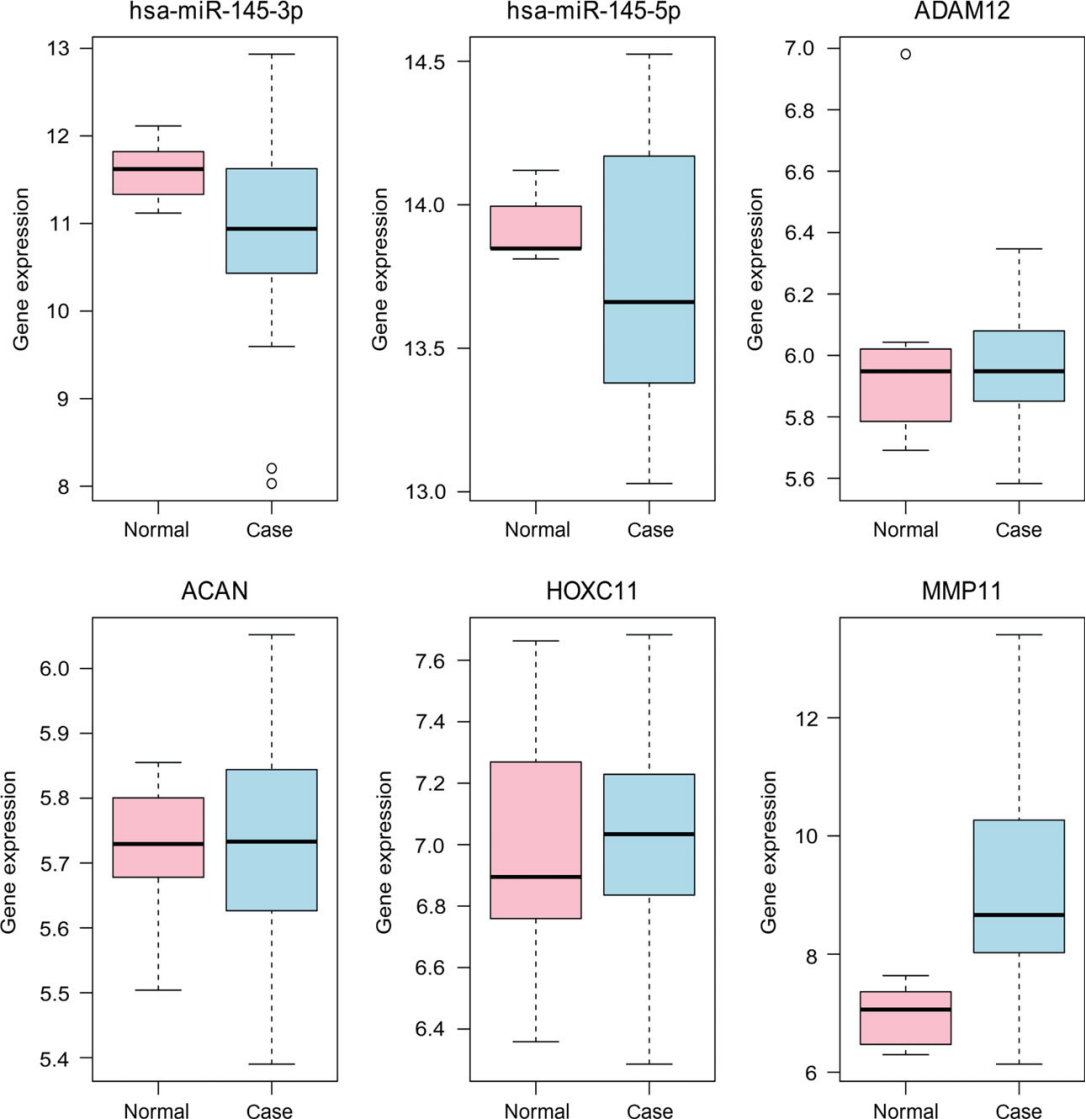
Validation of the expression levels of selected miRNAs and in STAD based on GEO database. The *x*‐axis shows the case and normal groups and the *y*‐axis shows expression read counts. Case group and normal group indicate STAD tissues and adjacent non‐tumor tissues.

In this study, two down‐regulated miRNAs (hsa‐miR‐145‐3p and hsa‐miR‐145‐5p) and four up‐regulated target genes (*ADAM12*,* ACAN*,* HOXC11* and *MMP11*) in STAD were selected to perform the expression validation (Fig. 8). Different expression levels of them between STAD and non‐tumor tissues were analyzed and depicted through box‐plots. These box‐plots were displayed visually by median and interquartile range. The expression levels of hsa‐miR‐145‐3p and hsa‐miR‐145‐5p were significantly down‐regulated in the case group compared with the normal group and the expression of *ADAM12*,* ACAN*,* HOXC11* and *MMP11* was significantly up‐regulated in the case group compared with the normal group. Compared with the normal group, the expression levels for the case group were consistent with our bioinformatics analysis.

### ROC curve analysis

**Figure 9:**
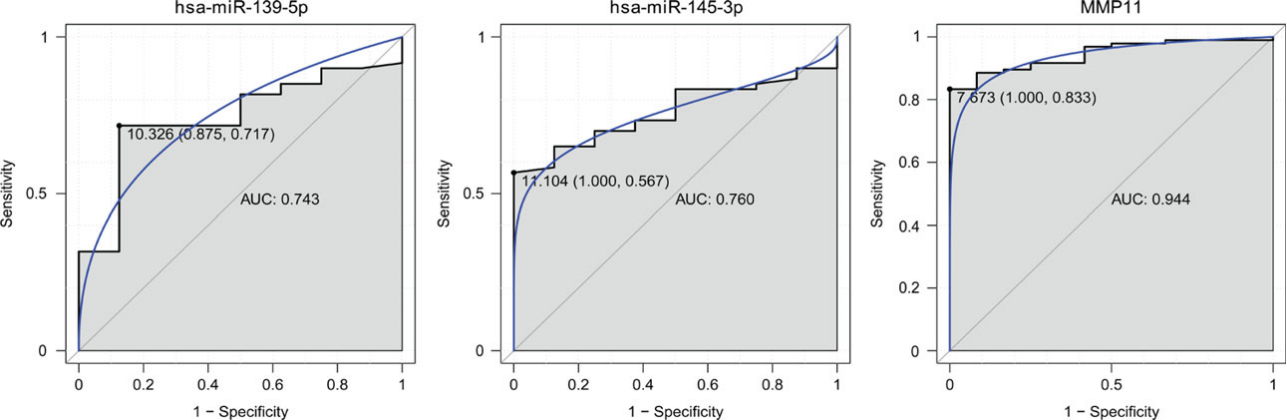
ROC curves of selected miRNAs and target genes between STAD patients and healthy controls. The ROC curves were used to show the diagnostic ability of these selected miRNAs and target genes with 1 – specificity (the proportion of false positives) and sensitivity (the proportion of true positives). The *x*‐axis shows 1 – specificity and the *y*‐axis shows sensitivity.

We performed ROC curve analyses and calculated the AUC to assess the discriminatory ability of two selected miRNAs (hsa‐miR‐139‐5p and hsa‐miR‐145‐3p) and one target gene (*MMP11*) from the GEO dataset (Fig. 9). The AUC for them was more than 0.7. *MMP11* had the largest AUC. For STAD diagnosis, the 1 – specificity (proportion of false positives) and sensitivity (proportion of true positives) of hsa‐miR‐139‐5p were 87.5% and 71.7%, respectively; the 1 – specificity (proportion of false positives) and sensitivity (proportion of true positives) of hsa‐miR‐145‐3p were 100% and 56.7%, respectively; the 1 – specificity (proportion of false positives) and sensitivity (proportion of true positives) of *MMP11* were 100% and 83.3%, respectively.

## Discussion

STAD remains the second leading cause of cancer death and makes up approximately 10% of newly diagnosed cancers 13. Therefore, an understanding of the molecular mechanism of STAD is needed. In the current study, we obtained nine differentially expressed miRNAs and 1248 DEGs for STAD. Additionally, target gene detection revealed 160 target genes of these differentially expressed miRNAs. After biological function analysis, we found that these target genes were most significantly enriched in the calcium signaling pathway and bile secretion.

Calcium signals can regulate the majority of physiological processes ranging from cell proliferation to cell apoptosis 14. It is reported that any disorders of Ca^2+^ channels and/or receptors will lead to various diseases, such as cancer 15. In this study, we found that the target genes (*SLC8A2*,* ADCY2*,* ATP2B4*,* CACNA1H*,* HTR2C*,* ATP2B2*,* PLN*,* NOS1* and *ATP2B3*) of differentially expressed miRNAs were associated with the calcium signaling pathway, which suggested a role for calcium signaling in STAD.

It is pointed out that there is a positive relationship between bile acid concentration and gastric carcinoma development, which suggests a carcinogenic role of bile acid in gastritis 16. Furthermore, Suzuki *et al*. 17 also found that patients with a high level of bile acid developed gastric carcinoma more frequently than those patients with a low bile acid level. In this study, we found that target genes (*ADCY2*,* AQP4*,* SLCO1A2* and *BAAT*) were related to bile secretion, which suggested a relationship between bile secretion and STAD.

All in all, these identified target genes played roles in the biological processes of calcium signaling and bile secretion in STAD. Additionally, we also investigated the function of all DEGs by KEGG pathway analysis and found that these DEGs were remarkably involved in signaling pathways of gastric acid secretion.

The principal secretory function of the stomach is secreting gastric acid 18. Gastric acid functions in a number of ways including modulating the gut microbiome, assisting in protein digestion and facilitating absorption of iron and calcium 19. It is reported that *H. pylori* is related to gastric carcinoma 20. Interestingly, it is found that in patients with relatives with gastric carcinoma, *H. pylori* infection is related to decreasing gastric acid secretion 21. In this study, we found all DEGs were significantly enriched in the signaling pathway of gastric acid secretion, which further demonstrated the role of gastric acid secretion in the development of STAD.

Among identified miRNAs, mature the form of hsa‐miR‐139‐5p, hsa‐miR‐145‐3p, hsa‐miR‐145‐5p and hsa‐miR‐490‐3p was validated by qRT‐PCR and the results were consistent with the bioinformatics results except for the expression of hsa‐miR‐139‐5p. The small sample size we used for qRT‐PCR may account for this inconsistency. It is pointed out that hsa‐miR‐139‐5p shows decreased expression associated with STAD 22, 23. Previous reports found that hsa‐miR‐145 was a potential tumor suppressor and hsa‐miR‐145‐3p and hsa‐miR‐145‐5p were down‐regulated in stomach carcinoma 24, 25, 26. Kuo *et al*. 22 demonstrated that hsa‐miR‐490‐3p was down‐regulated in STAD gastric carcinoma tissues. In this study, our results were in agreement with previous reports, which further demonstrated the role of these differentially expressed miRNAs in the process of STAD. Significantly, both hsa‐miR‐139‐5p and hsa‐miR‐145‐3p had a potential diagnostic value for STAD.

Additionally, we also validated the target genes (*ADAM12*,* ACAN*,* HOXC11* and *MMP11*) of these four miRNAs mentioned above, and their expression patterns were consistent with the bioinformatics results. ADAM12 is a complicated and multi‐domain protein that functions in cell proliferation and movement 27. Furthermore, it is strongly expressed in various cancers including stomach carcinoma 28, 29. That mutation of *ACAN* occurs in stomach carcinoma has been shown by comprehensive whole‐genome and transcriptome sequencing analysis 30. *HOXC11* is considered to be a novel potential oncogene with altered expression in stomach carcinoma pathogenesis by association analysis with candidate gene strategy 31. *MMP11* is known a marker of tumor invasion and metastasis 32. Moreover, previous reports detected the expression level of *MMP11* by microarray analysis and found that it was elevated in stomach carcinoma patients and quantitative polymerase chain reaction analysis also confirmed its up‐regulated expression 32, 33. Herein, up‐regulated expression of *ADAM12*,* ACAN*,* HOXC11* and *MMP11* may be involved in the pathology of STAD. Interestingly, *MMP11* was significantly associated with STAD diagnoses.

Besides the above validated differentially expressed miRNAs, we also found two miRNAs (hsa‐miR‐196b and hsa‐miR‐135b) were also differentially expressed in STAD. Moreover, hsa‐miR‐196 and hsa‐miR‐135 covered the most downstream target genes in the regulatory network between differentially expressed miRNAs and target genes. It has been demonstrated that the expression level of hsa‐miR‐196b is significantly higher in stomach carcinoma 34, 35. Additionally, overexpression of hsa‐miR‐196b is linked to stomach carcinoma and may be considered as a stomach carcinoma marker 36, 37. It was demonstrated that hsa‐miR‐135b is up‐regulated in stomach carcinoma tissues 38, 39. Based on the results obtained in several studies, hsa‐miR‐135b is reported as a potential biomarker of the intestinal‐type of STAD 40, 41, 42, 43. In our study, hsa‐miR‐196b and hsa‐miR‐135b were also up‐regulated, which was in line with previous reports. Further qRT‐PCR validation experiments for hsa‐miR‐196b and hsa‐miR‐135b are needed.

There are limitations to our study. Firstly, target site information for miRNA–mRNA pairs is needed for validation of the miRNA–mRNA interaction. Secondly, RNA‐seq and miRNA‐seq are further needed to screen larger numbers of candidate miRNA and mRNA. Thirdly, a luciferase assay for direct verification of the identified miRNA–target interactions is needed in further studies.

## Conclusions

All in all, we found four key differentially expressed miRNAs including hsa‐miR‐139‐5p, hsa‐miR‐145‐3p, hsa‐miR‐145‐5p and hsa‐miR‐490‐3p, four DEGs (*ADAM12*,* ACAN*,* HOXC11* and *MMP11*) and three related signaling pathways (calcium signaling pathway, bile secretion and gastric acid secretion) in STAD, which provide a novel field for understanding the pathological mechanism of STAD. In addition, hsa‐miR‐139‐5p, hsa‐miR‐145‐3p and *MMP11* have a potential diagnostic value for STAD.

## Author contributions

The study was designed by LZ and HT; the grant receiver was LZ; the experiments were conducted by YS; the manuscript was written by JL and FL.

## Supporting information


**Fig. S1.** Venn diagram of MTIs in the groups of miRTarBAse database *vs* miRWalk database.

**Table S1.** The original miR–target interactions from the miRWalk database.

**Table S2.** The original miR–target interactions from the miRTarBAse database.
