# Supplementary figures and images for: Identification of key miRNAs and genes associated with stomach adenocarcinoma from The Cancer Genome Atlas database
Source: FEBS Open Bio. 2018 Jan 2;8(2):279–94. doi: 10.1002/2211-5463.12365 (PMC5794471; doi:10.1002/2211-5463.12365)

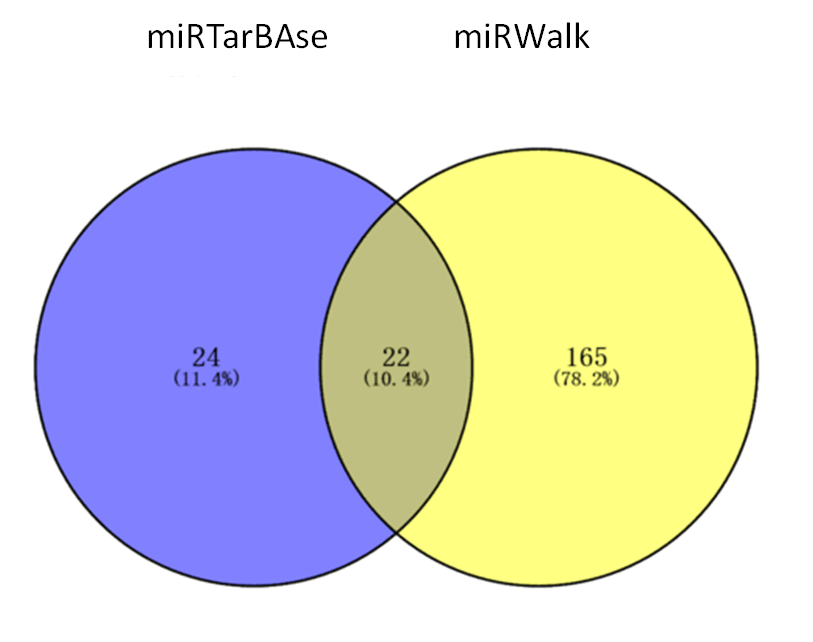

Supplement: Supplementary file 1 — Fig. S1. Venn diagram of MTIs in the groups of miRTarBAse database vs miRWalk database. [file FEB4-8-279-s001.tif]
